# Value‐added probiotic development by high‐solid fermentation of sweet potato with *Saccharomyces boulardii*

**DOI:** 10.1002/fsn3.441

**Published:** 2016-12-09

**Authors:** Carmen Campbell, Ananda K. Nanjundaswamy, Victor Njiti, Qun Xia, Franklin Chukwuma

**Affiliations:** ^1^ Department of AgricultureSchool of Agriculture, Research, Extension and Applied SciencesAlcorn State UniversityLormanMSUSA

**Keywords:** amino acid, CFU, fatty acid, fermented, *Ipomoea batatas*, neutral detergent fiber, viable cell

## Abstract

Controlled fermentation of Sweet potato (*Ipomoea batatas*) var. Beauregard by yeast, *Saccharomyces boulardii* (MAY 796) to enhance the nutritional value of sweet potato was investigated. An average 8.00 × 10^10^ Colony Forming Units (CFU)/g of viable cells were obtained over 5‐day high‐solid fermentation. Yeast cell viability did not change significantly over time at 4°C whereas the number of viable yeast cells reduced significantly at room temperature (25°C), which was approximately 40% in 12 months. Overall, the controlled fermentation of sweet potato by MAY 796 enhanced protein, crude fiber, neutral detergent fiber, acid detergent fiber, amino acid, and fatty acid levels. Development of value‐added sweet potato has a great potential in animal feed and human nutrition. *S. boulardii*‐ fermented sweet potato has great potential as probiotic‐enriched animal feed and/or functional food for human nutrition.

## 1 Introduction

Sweet Potato (*Ipomoea batatas* (L.) Lam) is an important crop of southern United States due to the ideal long frost‐free growing season. Globally, sweet potato is ranked at the seventh position next to rice, wheat, corn, and cassava consumption. This is primarily due to the versatile adaptation of the crop to diverse environmental and climatic conditions. Sweet potato is considered as a vegetable crop and has various food and feed applications. According to the USDA‐NASS (2015), the U.S. production topped 29 million hundredweight (cwt). Southern states dominated in production with North Carolina and California ranking top states followed by Texas and Mississippi. Sweet potatoes are rich in wide range of nutrients which include dietary fiber, vitamins, phenolic compounds, and carotenes (Ray & Tomlins, 2010). Sweet potato has been used as animal feed in poultry, pigs, and in livestock (Chittaranjan, 2007; Lebot, 2009; Woolfe, 1992). On dry basis, sweet potato is known to contain about 80% starch and a potential feedstock for bioethanol production (Santa‐Maria, Yencho, Haigler, & Sosinski, 2011).

Fermentation of vegetable products with beneficial microbes such as *Lactobacillus* (LA)*, Sacchromyces, Bacillus*, etc. have been carried out for preservation and nutritional enhancement (Karovičova, Grief, Kohajdova, & Hybenova, 2001). Salt‐incorporated fermentation of cabbage, cucumber, and olives are commercially available which not only provide preservation but also enhance the nutritional quality (Maifreni, Marino, & Conte, 2004). Some of the Asian LA‐fermented vegetables such as radish, mustard, and cauliflowers are known to enhance flavor and preserve some biochemicals such as carotenes and phenolics (Shivashankara, Isobe, Al‐Haq, Takenaka, & Shiina, 2004). Because of the starch‐rich nature of sweet potato and its co‐products from processing industry, various fermentation routes have been used to develop value‐added products (Ray & Ward, 2006). Ray, Panda, Swain, and Sivakumar (2012) developed wine from anthocyanin‐rich sweet potato by fermentation of *Saccharomyces cerevisiae*. The resultant wine showed a 58.95% anti‐oxidant activity with a similar pH, tannin, and phenol profile of grape wine. Pagana et al. (2014) used organic waste from sweet potato processing for canning as raw material for production of lactic acid, using *Lactobacillus rhamnosus* with yield of 10 g/l of lactic acid production in 72 hr. Additionally, sweet potato and its waste has been used to produce antibiotic such as tetracycline (Yang and Yuan, 1989), citric acid, lactic acid and ethanol (Ray & Palaniswami, 2008; Wongkhalaung, 1995).

Food‐ and feed‐borne gastroenteritis are common among humans and animals. Some of the pathogens to blame for these outbreaks are *Salmonella*,* E. coli*, and *Clostridium difficile* to name a few major pathogens (Govaris, Solomakos, Pexara, & Chatzopoulou, 2010). These gastrointestinal disorders can be cured by use of viable microorganisms called “probiotics”. Some of the well‐known prokaryotic probiotic genera of microorganism are *Lactobacillus*,* Bifidobacterium*,* Enterococcus*,* Oenoccoccus*,* Propionibacterium*,* Bacillus*, and *Clostridium butyricum* (Gibson, 1998). Only a few eukaryotic microbes such as *Saccharomyces cerevisiae* and *Saccharomyces boulardii* are known to exhibit probiotic effect in humans and animals. They have been extensively studied for their ability in reducing gastrointestinal inflammation and other disorders (Hamedi et al., 2013; Hudson et al., 2014; Pothoulakis, 2009). *Saccharomyces boulardii* is known to act by providing receptor sites for binding some toxins produced by pathogens. Yeast glucans and mannans are also known to play a critical role in the toxin‐binding process. These actions are well mediated at different physiological conditions including anaerobic stress, low pH, and osmotic shocks (Klis, Boorsma, & DeGroot, 2006; Lesage & Bussey, 2006; Orlean, 2012); *S. boulardii* can induce immune response by increasing the IgA and IgG levels (Lauren et al. 2016; Matar et al., 2015; Kim et al., 2013; Buts, Bernasconi, Vaerman, & Dive, 1990; Qamar et al., 2001; Rodrigues et al., 2000); and *S. boulardii* proteases are known to inhibit Toxin A and Toxin B of *Clostridium difficile*, which are mainly responsible for diarrhea and colitis (Castagliuolo, Riegler, Valenick, LaMont, & Pothoulakis, 1999). Due to the probiotic nature of *S. boulardii*, attempts have been made to ferment various food products and agricultural produce to provide supplemental probiotic for humans as well as for animals. For example Fratianni et al. (2013) used berry juice as a medium to grow yeast and used alginate‐inulin‐xanthan gum microencapsulation for better delivery and enhancement of cell viability. They reported a healthy growth of 7.59 × 10^10^ CFU/ml viable cells when stored for a month at 4°C. Many other co‐products of food industry such as rice bran (Ryan et al., 2011), raw wheat (Nguyen & Herve, 1997) and , tomato juice (Fratianni et al., 2013) have been used for fermenting *S. boulardii*. With the abundant supply of sweet potato in southern United States, production of value‐added products provides an opportunity to diversify sweet potato uses. Keeping this in mind, sweet potato was used a feedstock for production of *S. boulardii*‐enriched fermented product. The enriched product can serve as potential probiotic in animal feed supplement to control gastrointestinal disorders and also have application in human health and nutrition. The overall objective of this study was to develop *S. boulardii*‐fermented sweet potato and characterize the viable cell counts over time and nutritional profiling of the end product (Table 1).

**Table 1:** Viable cell counts at different time points

Months	4°C	25°C
0	7.9 × 10^10 a^	8.5 × 10^10 a^
4	8.0 × 10^10 a^	7.3 × 10^10 a^
8	7.9 × 10^10 a^	6.9 × 10^10 b^
12	7.7 × 10^10 a^	5.9 × 10^10 b^

Counts are average of two replicates.

## 2 Materials and Methods

### 2.1 Microbial culture

Lyophilized cultures of *S. boulardii* (MAY 796) were obtained from American Type Culture Collection (ATCC, Manassas, VA), revived on potato dextrose agar (PDA) and stored at 4°C for 10 days. After revival, cultures were inoculated into yeast extract malt extract broth and incubated at 30°C on an orbital shaker at 300 rpm for 5 days and stored in a deep freezer at −80°C.

### 2.2 Inoculum generation

About one vial (1 ml) from – 80°C storage was thawed and was inoculated into potato dextrose broth which was sterilized at 121°C for 30 min. Inoculum was grown at 30°C on an orbital shaker at 200 rpm for 48 hr.

### 2.3 Media preparation for high solid fermentation

“Beauregard” sweet potato was collected from Mound Bayou, MS, and was stored at room temperature at Alcorn State University Experiment Station, Lorman, MS. Selected sweet potatoes were washed thoroughly with running water to remove any surface debris, was chopped and placed in a drying chamber for 3 days at 28°C. Sweet potato flour was prepared by milling in an Udy bench top grinder. About 60 g of the finely ground sweet potato flour was added to 1000 ml flask with 300 ml of H_2_O. Other inorganic salts included were 1 g KH_2_PO_4_, 0.5 g MgSO_4_, and 0.5 g MnSO_4_ and ZnSO_4_ per liter. Samples were autoclaved at 121°C for 30 min. Upon cooling, flasks were inoculated with 10 ml of MAY 796. This production media was then placed in an incubator shaker at 300 rpm for 5 days at 30°C. Duplicate samples were prepared for statistical analysis.

### 2.4 Fermentation conditions

Flasks were inoculated and incubated at 30°C, 300 rpm for 5 days. Control flasks without inoculum were also maintained. Two replicates per treatment were employed. Samples were freeze dried for 48 h and stored at −80°C until further analyses.

### 2.5 Determination of viable cells of *S. boulardii*

Serial dilution technique was used to determine CFU which represents viable cells per gram of the final fermented samples. In brief, a serial dilution method was used and spread plate method of culturing yeast cells on PDA medium was carried out. Sample dilutions up to 10^−12^ were carried out. After incubation of plates at 30°C for 4 days, colonies from plates with the highest dilutions (where the colony numbers varied between 50 and 100) were counted and CFU/g of fermented samples was expressed.

### 2.6 Stability of the viable yeast cells

Samples were stored in Zip‐lock bags at 4° and 25°C (Room Temperature) and periodically (every 4 months) subjected for determination of viability, using serial dilution technique.

### 2.7 Nutritional profiling

Known quantity of freeze‐dried samples were weighed and sent to the Agricultural Experiment Station Chemical Laboratories (AESCL) at the University of Missouri‐Columbia for biochemical compositional analyses, using the ASOS methods as described in Nanjundaswamy and Vadlani (2011). Nutrition composition analyses included total amino acid profile, total fatty acid profile, crude fat and protein, crude fiber, % neutral detergent fiber (NDF), and % acid detergent fiber (ADF).

### 2.8 Statistical analyses

Data were analyzed by Statistical Analysis Software (SAS) version 9.2. Analysis of Variance was measured, using PROC ANOVA and Tukey test was used for pair‐wise comparisons. Significance was set at *p* < .05.

## 3 Results and Discussion

### 3.1 Viable cell count in fermented product

The end of fermentation viable cell count for *S. boulardii* was about 8.0 × 10^10^ CFU/g (Table 1). This cell viability count was similar to other studies (Fratianni et al., 2014; Torija, Rozès, Poblet, Guillamèn, & Mas, 2002). When the fermented product was stored at room temperature (25°C) and at 4°C, there was a decrease in the CFU over the next 12 months. The decrease in the CFU was not significant at 4°C (*p = *.8362), whereas the CFU decrease at room temperature was significant (*p = *.0032), which was about 44%. This information is very vital, since cell viability is critical for probiotic efficiency of the fermented‐product. Some of the earlier yeast cell stability studies conducted in liquid medium showed clear drop in the CFU over time due to the inherent metabolic stress, ethanol production, and decrease in membrane functionality (Casey, Magnus, & Ingledew, 1984; Nagodawithana, Castellano, & Steinkraus, 1974; Ough, 1966). It is plausible that the removal of water and reducing the water activity has a great impact on long‐term stability of the probiotic product. Also storage of the product at 4°C prevents any loss of viability of the probiotic.

### 3.2 Overall nutritional profile

**Figure 1:**
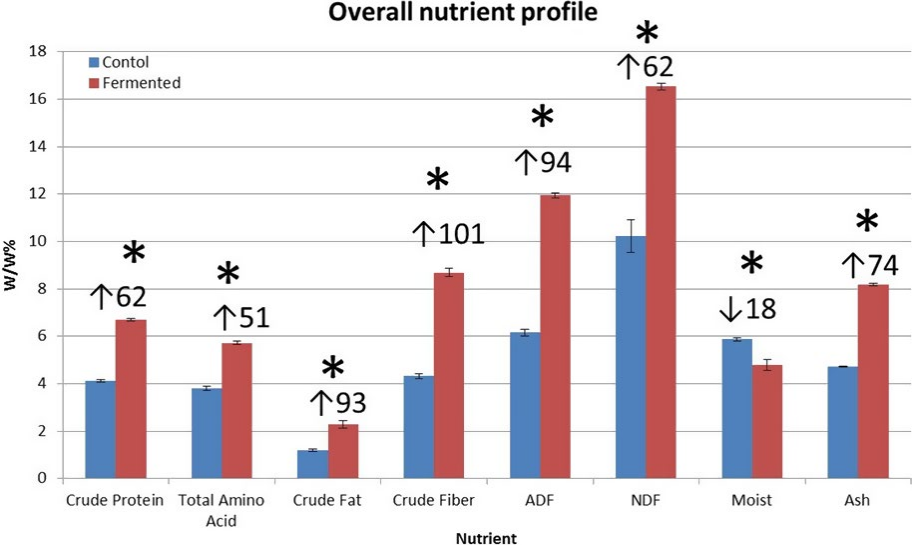
Nutrition composition. Means and standard errors are provided. *significantly different (*p* < .05) treatment from control. ↑% increase, ↓%decrease compared to control

The overall nutritional profile of fermented sweet potato *by S. bouldardii* is shown in Figure 1. Fermentation significantly (*p* ≤ .05) enhanced the % crude protein, total amino acid content, % crude fat, % crude fiber, % ADF, % NDF, and ash content compared to the control. Moisture was significantly greater (*p* ≤ .05) in the control than in the fermented sample. Actively growing yeast produced significant protein which contributed to the enhanced levels of total protein. A similar trend was observed in red yeast fermentation of dry distillers grain with solubles (DDGS), a co‐product of corn ethanol production (Nanjundaswamy & Vadlani, 2011). Endo‐ and exo‐proteases in the actively growing yeast can break down proteins into numerous amino acids, resulting in enhanced total amino acids (Sturley & Young, 2013). Neutral detergent fiber represents the total plant fiber or cell wall, including hemicellulose, cellulose, and lignin. Increased NDF results in higher digestible energy.

### 3.3 Amino acid profile of control and fermented sweet potato

**Figure 2:**
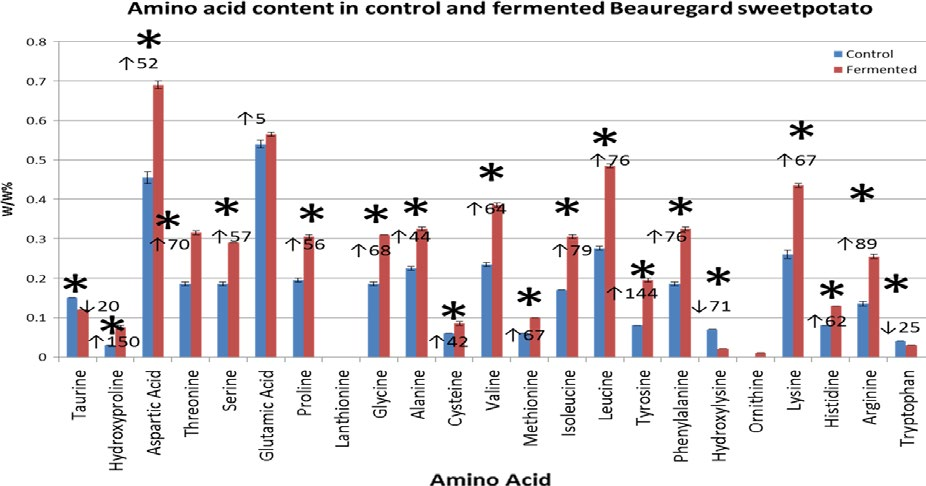
Amino acid Content. Means and standard errors are provided. *significantly different treatment from control (*p* < .05). ↑% increase, ↓%decrease compared to control

Compared to the control, fermented samples had significantly lower (*p *≤ .05) Taurine, hydroxylysine, and tryptophan (Figure 2). For all other amino acids, fermented samples had significantly higher (*p* ≤ .05) content than control, except for glutamic acid which was greater than control but not statistically significant. Overall, total amino acid content was significantly greater (*p* ≤ .05) in fermented samples than control.

Some of the important amino acids such as lysine, leucine, glutamic acid, and aspartic acid levels were higher in fermented samples compared to control. These amino acids are of importance in animal feed. The lysine levels of the fermented sweet potato is comparable to DDGS (Stein et al. 2005; Pahm, Hoehler, Pedersen, Simon, & Stein, 2006; Pahm, Pedersen, & Stein, 2006; Stein, Pedersen, Gibson, & Boersma, 2006; Urriola et al., 2009).

### 3.4 Fatty acid profile of control and fermented sweet potato

**Figure 3:**
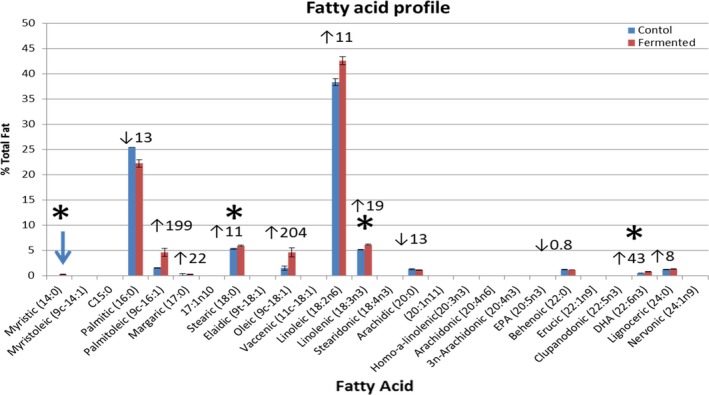
Fatty acid profile. Means and standard errors are provided. At *p* < .05, treatments were significantly different only for myristci acid, stearic acid, linolenic acid, and DHA. ↑% increase, ↓%decrease compared to control

Figure 3 represents fatty acid profile of fermented and control samples. At least 12 fatty acids were detected. Fermented samples had significantly higher (*p* ≤ .05) levels of myristic acid, stearic acid, linolenic acid, and DHA compared to the control. Of the remaining nine fatty acids, only six fatty acids were higher in content in fermented samples than control, but were not statistically significant; three fatty acids namely palmitic (16:0), stearidonic acid, and behnoic acid content were greater in control than fermented samples, but not statistically significant. A similar fatty acid profile was reported in wine from fermentation of different species of *Saccharomyces* under different temperatures (Torija et al., 2002). A very limited metabolic profiling has been reported with respect to fatty acid profile of *S. boulardii*. Ryan et al. (2011) provided insights into the metabolomics of rice bran‐fermented with *S. boulardii*. The *S. boulardii* fermentation undoubtedly changed the metabolomic profile for rice bran. GC‐MS profiling of the volatile fatty acid was positively influenced by fermentation. Fermented rice bran showed elevated levels of secondary metabolites such as ferulic acid.

## 4 Conclusions

High‐solid submerged fermentation of sweet potato with *S. boulardii* was successfully carried out with an end of fermentation viable cell count of 8.0 × 10^10^ CFU/g. The freeze‐dried fermented product showed good stability with respect to CFU at 4°C for 12 months. The overall nutritional profile of the fermented product was positively changed with increased total protein, total amino acid and NDF. *S. boulardii* value‐added sweet potato opens up new opportunities for value‐added probiotics for animal feed and human nutrition.

## Conflict of Interest

None declared.
